# Interferon regulatory factor-1 activates autophagy to aggravate hepatic ischemia-reperfusion injury via the P38/P62 pathway in mice

**DOI:** 10.1038/srep43684

**Published:** 2017-03-07

**Authors:** Yao Yu, Shipeng Li, Zhen Wang, Jindan He, Yijie Ding, Haiming Zhang, Wenli Yu, Yiwei Shi, Zilin Cui, Ximo Wang, Zhiliang Wang, Liying Sun, Rongxin Zhang, Hongyin Du, Zhijun Zhu

**Affiliations:** 1 First Central Clinical College, Tianjin Medical University, Tianjin 300192, China; 2 Key Laboratory of Organ Transplantation of Tianjin, Tianjin 300071, China; 3 Oriental Organ Transplant Center, Tianjin First Central Hospital, Tianjin 300192, China; 4 Tianjin Nankai Hospital, Tianjin 300100, China; 5 Affiliated Hospital of Logistics University of Chinese People’s Armed Police Forces, Tianjin 300162, China; 6 Beijing Friendship Hospital, Capital Medical University, Beijing 100050, China; 7 Department of Immunology and Inflammation, Tianjin Medical University, Tianjin 300070, China

## Abstract

Increasing evidence has linked autophagy to a detrimental role in hepatic ischemia- reperfusion (IR) injury (IRI). Here we focus on the role of interferon regulatory factor-1 (IRF-1) in regulating autophagy to aggravate hepatic IRI. We found that IRF-1 was up-regulated during hepatic IRI and was associated with an activation of the autophagic signaling. This increased IRF-1 expression, which was allied with high autophagic activity, amplified liver damage to IR, an effect which was abrogated by IRF-1 depletion. Moreover, IRF-1 contributed to P38 induced autophagic and apoptotic cell death, that can play a key role in liver dysfunction. The levels of P62 mRNA and protein were increased when P38 was activated and decreased when P38 was inhibited by SB203580. We conclude that IRF-1 functioned as a trigger to activate autophagy via P38 activation and that P62 was required for this P38-mediated autophagy. IRF-1 appears to exert a pivotal role in hepatic IRI, by predisposing hepatocytes to activate an autophagic pathway. Such an effect promotes autophagic cell death through the P38/P62 pathway. The identification of this novel pathway, that links expression levels of IRF-1 with autophagy, may provide new insights for the generation of novel protective therapies directed against hepatic IRI.

Hepatic ischemia-reperfusion (IR) injury (IRI), a condition in which hypoxia is accentuated following the ensuing reperfusion of blood flow and oxygen delivery, is an inevitable complication associated with liver transplantation, partial hepatectomy and hypovolemic shock^1^. This pathogenesis predisposes grafts to both short- and long-term dysfunction and contributes to poor prognosis and patient survival^2^,^3^. Many attempts have been undertaken to ameliorate hepatic IRI, including ischemia preconditioning, pharmacological and surgical manipulations and gene therapies^4^. However, despite advances in the management of grafts, the mechanisms of IRI remain largely unknown.

Autophagy is a highly conserved intracellular process in which impaired proteins or organelles are engulfed by double-membraned autophagosomes and transported to lysosomes for degradation^5^,^6^. The presence of basal levels of autophagy ensures that cells are capable of digesting cytoplasmic materials to meet energetic demands in stressful conditions such as starvation or hypoxia^7^,^8^. However, undisciplined autophagy, which can occur under extreme conditions such as following acute organ injury or reperfusion insult, may produce an accumulation of autophagic vacuoles, which may then predispose the cells to death^9^,^10^. It has been hypothesized that excessive autophagy, due to its relatively non-specific degradative actions, may devour organelles that are essential to protect against cell failure^11^. Of particular relevance to the present report are the findings suggesting that increased autophagy may lead to an increased susceptibility of murine livers to IRI^12^. Such findings indicate a potentially novel strategy to ameliorate the effects of IRI, as achieved by modulating levels of autophagy.

Interferon regulatory factor-1 (IRF-1) is one of a family of highly conserved transcriptional factors that regulates the expression of certain genes involved in innate and acquired immunity. Tsung, *et al*. in 2006 reported the first study of IRF-1 as related to hepatic IRI and found that IRF-1 exerts a detrimental role in hepatic IRI by modulating the expression of multiple inflammatory mediators^13^. The significance of these findings was the identification of a novel target for protection of livers against IRI. Originally identified as a transcriptional activator of IFN-β and IFN-α genes^14^,^15^, IRF-1 was subsequently demonstrated to play a critical role in the development of IRI^16^,^17^. Complementing these findings were results from IRF-1 gene knockout mice, which showed their livers to be protected from IRI^18^.

It has been demonstrated that the phosphorylation of P38, JNK and ERK1/2 may be downstream of the IRF-1 signaling pathway in the activation of processes promoting immune maturation and function of dendritic cells^19^. Related to this topic are the findings that activation of P38-MAPK was involved in the TNF-α and IFN-γ induced transcriptional activation of the iNOS gene, which consequently led to an increased activation of IRF-1^20^. Additionally, IFN-γ-mediated autophagy via P38-MAPK contributes to the ability of macrophages to kill intracellular bacteria and this macrophage activity is attenuated when treated with P38-MAPK inhibitor, SB203580. Such findings demonstrate that P38-MAPK-mediated autophagy can support IFN-γ-mediated cell-autonomous innate immunity^21^. Moreover, treatment with the P38 inhibitor, SB203580, substantially reduced LPS-induced P62 mRNA expression and LC3II accumulation suggesting a role for P38 in TLR4-mediated induction of P62 and autophagic substrate formation of macrophages in response to pathogen recognition^22^. Such a notion is supported by data showing an accumulation of P62 in cells exposed to chemically-induced oxidative-stress conditions^23^. With regard to the present report, increasing evidence has been presented which suggests that the P38 signaling pathway contributes to hepatic IRI, including processes leading to the induction of inflammatory cytokines such as IL-1β and TNF-α^24^,^25^. However the underlying mechanisms, including a link to autophagy in hepatic IRI, are not well defined. In specific, whether IRF-1 participates in IRI processes involving P38-induced autophagy is unclear. To address this issue, we investigated the relationship between IRF-1 expression and P38 in murine hepatic IRI. We hypothesized that IRF-1 functioned as a mediator involved with deteriorating hepatic IRI via up-regulation of P38 activation. Such an effect would subsequently result in the activation of the P62 pathway thereby contributing to autophagic responses in IRI.

## Results

### Livers exposed to IR show severe damage

**Figure 1:**
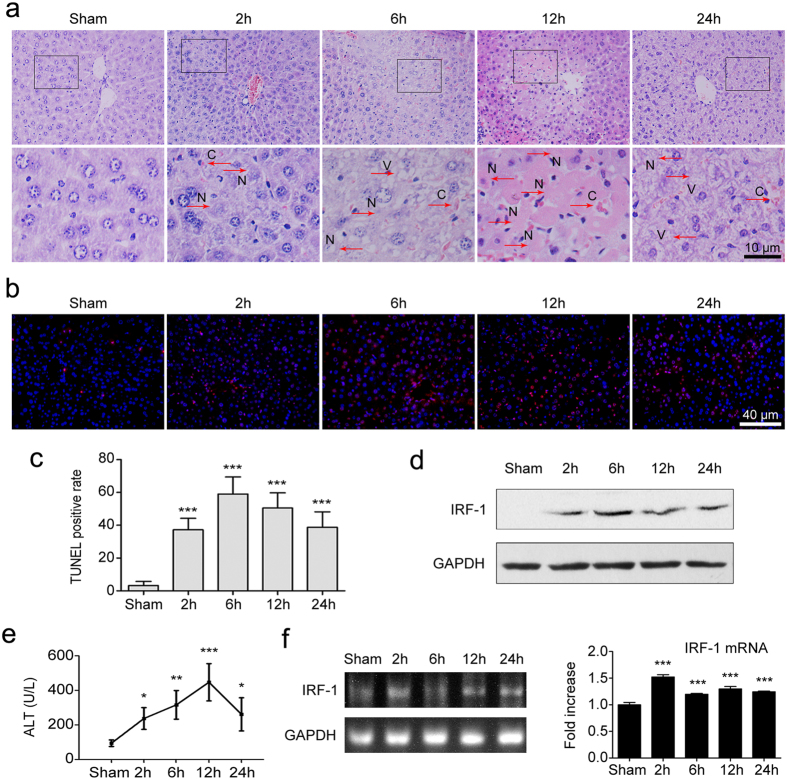
Hepatic inflow occlusion and reflow induced hepatic IRF-1 up-regulation and hepatic damage. (**a**) Representative histopathological images of livers after exposure to different times of reperfusion (2, 6, 12, or 24 h). H&E staining. Red arrows indicate typical changes of vacuolation (V), sinusoidal congestion (C) and hepatocyte necrosis (N). (**b**,**c**) Apoptosis as assessed by TUNEL assay in livers as described in (**a**). (**d**) Western blot analysis of IRF-1 levels in livers treated as described in (**a**). (**e**) The serum ALT levels were significantly increased in mice after exposure to IR as compared with the sham group. (**f**) Relative expression of IRF-1 mRNA in livers as determined using qRT-PCR and expressed as fold change over that of the sham group. *P < 0.05, **P < 0.01 and ***P < 0.001.

Mice were randomly divided into sham or IR treatment groups. As shown in Fig. 1a, the histology of livers revealed extensive areas of sinusoidal congestion and ballooning degeneration after exposure to IR. Severe sinusoidal stenosis and patchy necrosis were present within 12 h after reperfusion. Consistently, serum ALT levels increased significantly (*P* < 0.05) and peaked at 12 h after reperfusion as compared with that of the sham group (Fig. 1e). TUNEL assay revealed highly increased (*P* < 0.001) hepatocellular apoptotic rates in livers subjected to IR, which was rarely seen in the sham group (Fig. 1b,c).

### IRF-1 is up-regulated during hepatic IRI

To determine whether this injury was associated with changes in IRF-1 expression, we examined levels of endogenous hepatic IRF-1 mRNA and protein expression. As shown in Fig. 1f, the levels of hepatic IRF-1 mRNA promptly increased (*P* < 0.001) after reperfusion, peaked at 2 h and were maintained at high levels until 24 h after reperfusion. IRF-1 protein was also up-regulated in livers after exposure to IR, with these reaching maximal levels at 6 h after reperfusion (Fig. 1d). These results clearly implicated IRF-1 as a mediator of liver damage during the process of hepatic IRI.

### IR triggers autophagic responses in murine livers

**Figure 2:**
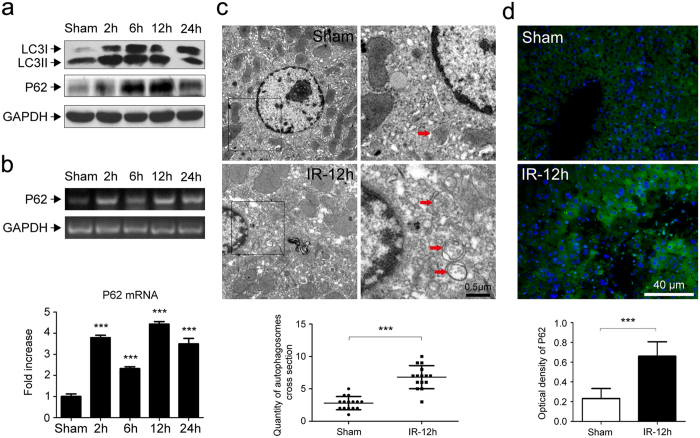
Changes of autophagic signaling in livers after exposure to IR. (**a**) Western blot analysis of autophagic marker (LC3II and P62) levels after exposure to different times of reperfusion (2, 6, 12 or 24 h). (**b**) Expression of P62 mRNA in livers after exposure to IR, expressed as fold change over that of the sham group, and determined using qRT-PCR. (**c**) Quantitative changes of autophagic vacuoles under transmission electron microscopy (TEM) in livers after exposure to 12 h-reperfusion. Typical images are shown at a higher magnification. (**d**) Immunofluorescent staining and quantitative changes of P62 (green) in livers exposed to 12 h- reperfusion. The nucleus was counter-stained with DAPI (blue). ***P < 0.001.

Western blot assay was utilized to examine the expression of LC3II (a key marker of autophagy) and P62 (a key substrate of autophagy) in livers. As shown in Fig. 2a, the expression of LC3II was aberrantly up-regulated in livers after exposure to IR-2-12 h and returned normal at 24 h after IR. The expression of P62 was up-regulated in livers after exposure to IR-2-24 h. To evaluate whether this escalation in P62 was the result of increased P62 transcription, we quantified P62 mRNA levels using qRT-PCR. P62 mRNA increased more than 2 fold in livers after exposure to IR (*P* < 0.001, Fig. 2b), which in combination with the demonstration of a P62 protein accumulation clearly show an activated autophagic capability after IR exposure. Next, we imaged autophagic vacuoles with the use of transmission electron microscopy (TEM). The ultrastructure of IR livers displayed abundant autophagic vacuoles at 12 h after exposure, which was rarely seen in the sham group (*P* < 0.001, Fig. 2c). Similar to P62 results obtained with western blot, the immunofluorescence staining of P62 was also significantly increased (*P* < 0.001) in livers after reperfusion at 12 h as compared with that of the sham group (Fig. 2d).

### IRF-1 overexpression increases hepatic susceptibility to IRI

**Figure 3:**
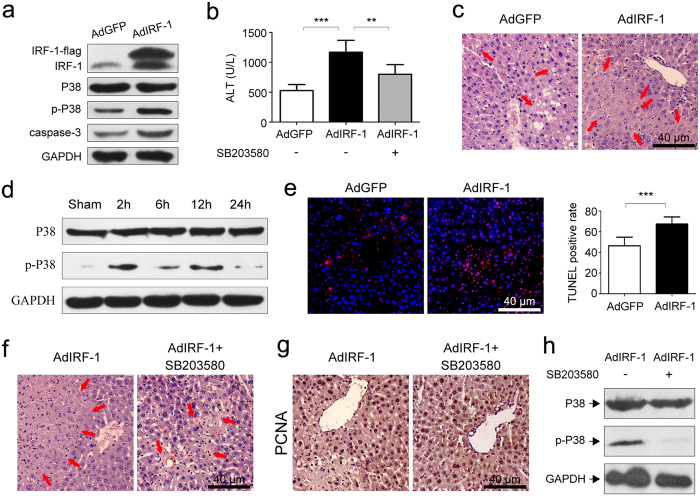
Increased IRF-1 facilitated P38 phosphorylation and aggravated liver injury, whereas SB203580 inhibited P38 activation and reduced liver injury. (**a**) Western blot analysis of IRF-1, P38, p-P38 and caspase-3 levels in livers from 12 h-IR mice pretreated with AdGFP (empty adenoviral vector control) or AdIRF-1 (adenoviruses encoding murine IRF-1). (**b**) AdIRF-1 pretreatment increased ALT levels, which were decreased by SB203580. (**c**) Representative histopathological images of livers as described in (**a**). H&E staining. (**d**) Western blot analysis of P38 and p-P38 levels in livers from control and IR (2, 6, 12, 24 h after reperfusion) mice. (**e**) Apoptosis was assessed by TUNEL assay in livers as described in (**a**). (**f**) Representative histopathological images of livers exposed to AdIRF-1 or AdIRF-1 plus SB203580. H&E staining. (**g**) Immunohistochemical staining of proliferating cell nuclear antigen (PCNA) in livers as described in (**f**). (**h**) Western blot analysis of P38 and p-P38 in livers as described in (**f**). **P < 0.01 and ***P < 0.001.

Up-regulated levels of IRF-1 observed during hepatic IR are believed to be involved in the pathogenesis of liver damage. To further evaluate the role of IRF-1 in hepatic IRI, adenovirus encoding murine IRF-1 (AdIRF-1) was administered to mice as an approach to mimic the IRI model at 12 h. Injection of AdIRF-1 via caudal vein resulted in an upsurge of IRF-1 expression (Fig. 3a). Minimal IRF-1 expression was observed in murine liver controls given equivalent plaque forming units of empty adenoviral vector (AdGFP). The results presented in Fig. 3 recapitulated the deduction that IRI may be aggravated by IRF-1 overexpression, as substantiated from the results of serum ALT levels and H&E staining. Specifically, mean levels of serum ALT were significantly increased (*P* < 0.001) in the AdIRF-1, compared with the AdGFP group (Fig. 3b). Results obtained from histological staining showed that increased IRF-1 expression tended to amplify hepatocyte necrosis as compared to livers treated with AdGFP (Fig. 3c).

### IRI induced by IRF-1 overexpression involves P38 activation

As p-P38 was found to increase in livers after exposure to IR (Fig. 3d), we sought to determine whether overexpression of IRF-1 would play a role in activating P38. While substantial increases in p-P38 were observed in AdIRF-1 as compared with AdGFP treated livers, no statistically significant differences in total P38 levels were obtained between these two treatments (Fig. 3a). TUNEL assay (Fig. 3e), which correlated positively with increased levels of caspase-3 expression (Fig. 3a), showed that IRF-1 overexpression may be associated with increased apoptotic hepatocellular death.

### The caustic role of IRF-1 in IRI is dependent on P38-MAPK

To further examine the role of P38 activation in liver damage, we examined the effect of the P38 inhibitor, SB203580, within the IR model pretreated with AdIRF-1. As shown in Fig. 3h, P38 activation was inhibited by SB203580 in the AdIRF-1 plus SB203580 as compared with the AdIRF-1 group. No obvious changes in total P38 expression were found in either group. IRI appeared to be alleviated with inhibition of P38 activation by SB203580 (Fig. 3f), which was associated with a significant decrease (*P* < 0.01) in serum ALT levels within the AdIRF-1 plus SB203580, as compared with the AdIRF-1 group (Fig. 3b). Consistent with this reduction in damage, liver repair was found to be increased in the AdIRF-1 plus SB203580 versus AdIRF-1 group, as evidenced by increased staining of PCNA in hepatocytes (Fig. 3g).

### Increased IRF-1 expression enhances hepatic autophagic responses to IR

**Figure 4:**
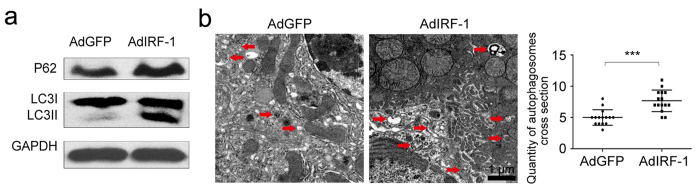
Effects of IRF-1 on autophagic signaling induced by IR. (**a**) Western blot analysis of LC3II and P62 levels in livers pretreated with AdGFP or AdIRF-1. (**b**) Quantitative changes in liver autophagic vacuoles imaged using TEM in livers as described in (**a**). ***P < 0.001.

AdIRF-1 pretreatment increased the levels of LC3II and P62 expression in livers after exposure to IR (Fig. 4a). In accord with these findings, images from TEM confirmed that an increased autophagic flux was present in AdIRF-1 livers as compared with the AdGFP (*P* < 0.001, Fig. 4b).

### Effects of IRF-1 deficiency on autophagic signaling and hepatic IRI

**Figure 5:**
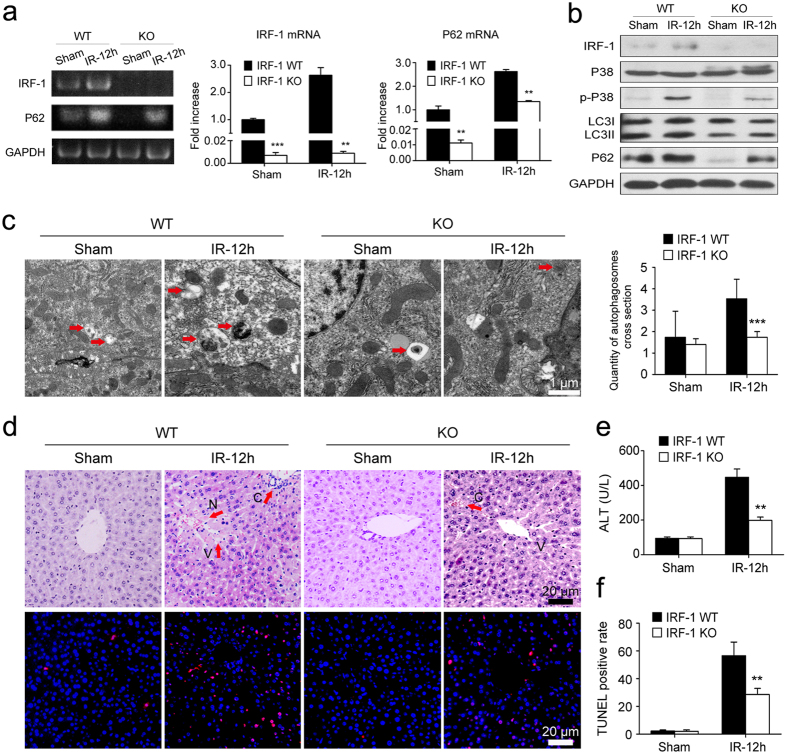
IRF-1 deficiency decreased autophagic signaling and ameliorated hepatic IRI. (**a**) Relative IRF-1 and P62 mRNA expression in IRF-1 wildtype (IRF-1 WT, IRF-1^+/+^) and IRF-1 knockout (IRF-1 KO, IRF-1^−/−^) murine livers as determined using qRT-PCR and expressed as fold change over that of sham controls. (**b**) Western blot analysis of IRF-1, P38, p-P38, LC3 and P62 levels in livers as described in (**a**). (**c**) Quantitative changes of autophagic vacuoles imaged using TEM in livers as described in (**a**). (**d**, **f**) Representative histopathological images and TUNEL staining of livers as described in (**a**). Red arrows indicate typical changes of vacuolation (V), sinusoidal congestion (C) and hepatocyte necrosis (N). (**e**) Mean levels of serum ALT in mice treated as described in (**a**). **P < 0.01 and ***P < 0.001.

To further determine the role of IRF-1 in hepatic IRI, IRF-1 knockout (IRF-1 KO, IRF-1^−/−^) mice^18^ were used to evaluate the changes in hepatic damage and autophagic signaling. As shown in Fig. 5a,b, IRF-1 mRNA and protein levels were down-regulated in IRF-1 KO mice as compared with the IRF-1 wildtype (IRF-1 WT, IRF-1^+/+^) mice. Results from TEM revealed a decrease in autophagic signaling, with bare autophagic vacuoles being present in livers of IRF-1 KO mice after exposure to IR (*P* < 0.001, Fig. 5c). This effect was associated with significantly decreased (*P* < 0.01) serum ALT levels in IRF-1 KO versus IRF-1 WT mice (Fig. 5e). IRI appeared to be ameliorated in IRF-1 KO mice as assessed by H&E and TUNEL staining. Reduced amounts of necrosis, sinusoidal congestion and ballooning degeneration were observed in the livers of IRF-1 KO versus IRF-1 WT mice after exposure to IR (Fig. 5d). In addition, the rate of hepatocellular apoptosis was significantly lower (*P* < 0.01) in the livers of IRF-1 KO mice exposed to IR (Fig. 5d,f). Collectively, these results indicate that IRF-1 may be involved in processes related to hepatic IRI, which are associated with induction of the autophagic signaling pathway.

### Regulatory effects of IRF-1 on autophagic responses to IR *in vitro*

The relationship between IRF-1 and autophagy was further evaluated in cultured AML12 cells. In these experiments, we employed IRF-1 overexpression and IRF-1 defection models of AML12 cells to examine quantitative changes in autophagic responses to IR *in vitro*. Hypoxia and re-oxygenation models were established to simulate the process of IR *in vitro*, IRF-1 was overexpressed in this model by propagating AdIRF-1 in AML12 cells and defected by IRF-1 siRNA transfection.

**Figure 6:**
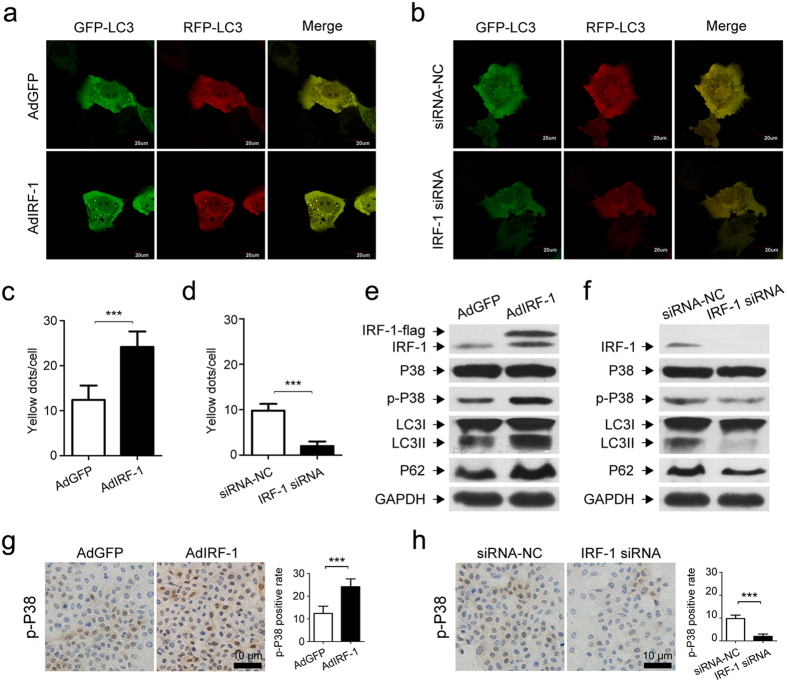
Effects of IRF-1 on autophagic and P38 signaling in AML12 cells induced by IR. (**a**,**b**) AML12 cells were transfected with AdIRF-1 or IRF-1 siRNA before being subjected to a simulated IR treatment. Confocal images of yellow (autophagosomes) and red (autolysosomes) puncta were collected from AML12 cells infected with GFP-RFP-LC3. (**c**,**d**) The data was quantified by counting the number of autophagosomes (yellow dots) per cell. (**e**,**f**) Western blot analysis of IRF-1, P38, p-P38, LC3II and P62 levels in AML12 cells as described in (**a**,**b**). (**g**,**h**) Immunocytochemical staining and quantitative changes of p-P38 in AML12 cells as described in (**a**,**b**). ***P < 0.001.

Two IRF-1 siRNAs, siRNA1 (S1) and siRNA2 (S2), as well as a negative control (NC) were selected for use in these experiments. After transfecting siRNAs into AML12 cells, IRF-1 expression was determined with use of western blot assay. We found that siRNA2 (S2) significantly inhibited IRF-1 expression and was subsequently used in our experiments. As shown in Fig. 6, the expression of LC3II and P62 were up-regulated in cells pretreated with AdIRF-1 (Fig. 6e), but down-regulated after treatment with IRF-1 siRNA (Fig. 6f), results which were verified by confocal microscopy. To determine the effects of IRF-1 overexpression on autophagosomes and autolysosomes formation, a mRFP-GFP tandem fluorescent-tagged LC3 construct was transfected into AML12 cells. As shown in Fig. 6a,b, some green and red puncta were clearly present after transfection. The number of both yellow and red dots were significantly increased (*P* < 0.001) after pretreatment with AdIRF-1 (Fig. 6a,c), indicating an increase in the formation of both autophagosomes and autolysosomes. Conversely, yellow and red dots were rarely seen in cells pretreated with IRF-1 siRNA as compared with that of siRNA-NC pretreatment condition (*P* < 0.001, Fig. 6b,d).

### IRF-1 induces autophagy via P38-MAPK activation

To verify the molecular mechanisms of IRF-1 induced autophagy in IRI, the P38-MAPK, which modulates the induction of autophagic signaling and apoptosis, was assessed. Results from the western blot assay were consistent with immunostaining in showing that AdIRF-1 pretreatment enhanced the phosphorylation of P38 (Fig. 6e,g). Conversely, down-regulation of IRF-1 decreased P38 phosphorylation (Fig. 6f,h).

### P62 is required for P38-mediated autophagic responses in AML12 cells

**Figure 7:**
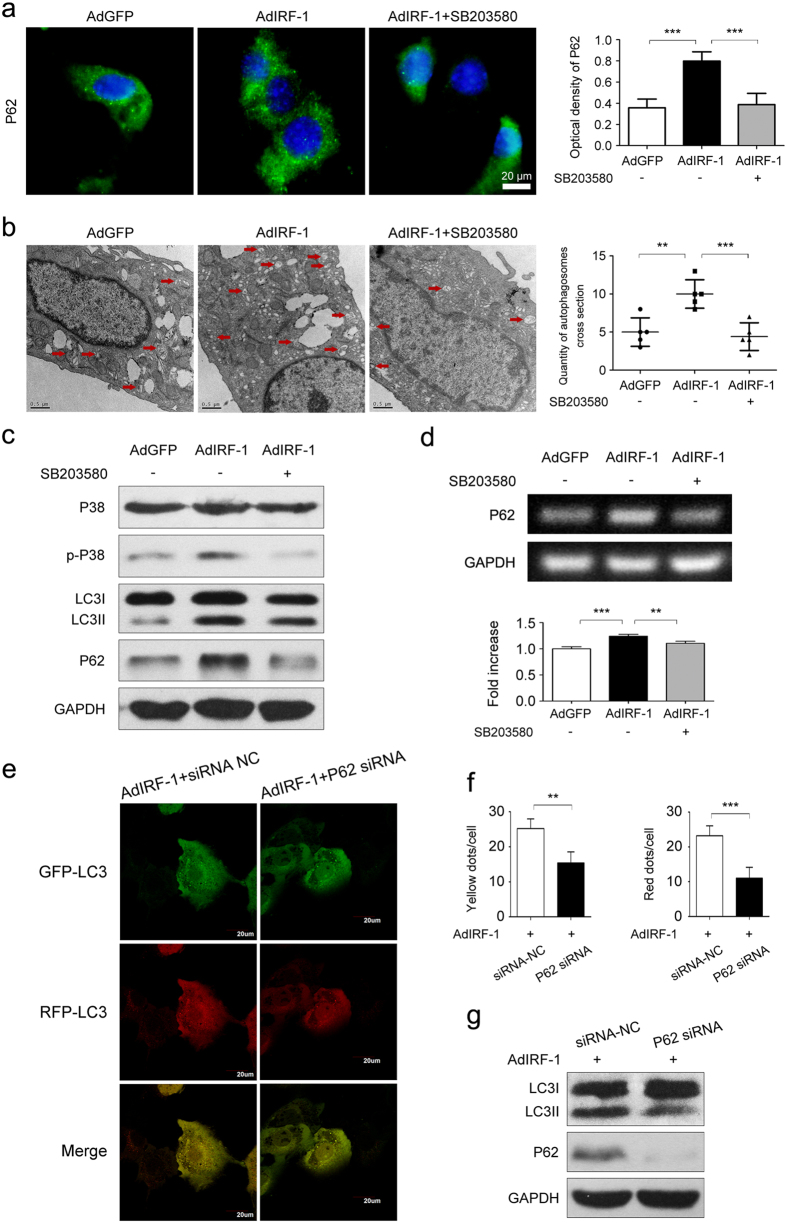
P38 inhibition decreased autophagic signaling by sequestering P62 in AML12 cells. (**a**) Immunofluorescence and quantitative changes of P62 (green) in AML12 cells after exposure to AdGFP, AdIRF-1 or AdIRF-1 plus SB203580. The nucleus was counter-stained with DAPI (blue). Scale bar: 10 μm. (**b**) Quantitative changes of autophagic vacuoles imaged using TEM in AML12 cells treated as described in (**a**). Scale bar: 0.5 um. (**c**) Western blot analysis of P38, p-P38, LC3II and P62 levels in AML12 cells treated as described in (**a**). (**d**) Relative expression of P62 mRNA in AML12 cells treated as described in (**a**), determined using qRT-PCR and expressed as fold change over that of AdGFP. (**e**) AML12 cells were transfected with AdIRF-1 or P62 siRNA before being subjected to a simulated IR treatment. Confocal images of yellow (autophagosomes) and red (autolysosomes) puncta were collected from AML12 cells infected with GFP-RFP-LC3. (**f**) The data was quantified by counting the number of autophagosomes (yellow dots) and autolysosomes (red dots) per cell. (**g**) Western blot analysis of LC3II and P62 levels in AML12 cells treated as described in (**e**). **P < 0.01 and ***P < 0.001.

To further investigate the role of P38 in IRF-1 enhanced autophagic responses, P38 activation was suppressed using SB203580. The resultant effects on P38-induced P62 dot formation as a marker of aggresome-like induced structures (ALIS) were then examined. As shown in Fig. 7c, the phosphorylation of P38 was blocked by SB203580, along with the inhibition of LC3II and P62 expression. Results from immunofluorescence staining of P62 revealed that abundant P62 puncta formation (green) were present in P38 activated cells which, by contrast, was rarely observed when P38 was inactivated (*P* < 0.001, Fig. 7a). The P38-induced P62 dots, which were confirmed by TEM images, indicated that puncta formation and recruitment of P62 into these complexes were regulated by P38 in these AML12 cells (*P* < 0.01, Fig. 7b). In addition, P62 mRNA was also significantly increased after IRF-1 induced P38 activation and this effect was inhibited by SB203580 (*P* < 0.01, Fig. 7d). Collectively, these results suggest that P38 transcriptionally up-regulates P62 expression levels, resulting in the formation of ALIS which are substrates for autophagy. Then P62 was defected by siRNA transfection. As shown in Fig. 7g, the expression of P62, as well as LC3II were decreased, along with a slight increase of LC3I expression. Confocal images showed that AML12 cells transfected with P62 siRNA displayed smaller amounts of both autophagosomes (yellow) and autolysosomes (red) formation (Fig. 7e,f).

## Discussion

Autophagy is regarded as a natural and essential defense mechanism against stress, inflammation and oncotherapy^26^, and has been implicated in the pathogenesis of numerous human diseases^27^. Increasing amounts of evidence have linked autophagy to IRI, but the exact mechanisms involved remain elusive. While it has been shown that ischemia insult activates autophagy to provide a protective role in myocardial ischemia, the intemperate activation of autophagy during reperfusion causes cell damage and eventually cell death^28^. In the present study, we found that IR exposure resulted in the accumulation of autophagic vacuoles accompanied by a dramatic increase in LC3II as demonstrated both *in vivo* and *in vitro*. However, the interpretation of such increases in autophagic vacuoles is somewhat equivocal as it could be due to an increase in the formation or a decrease in the clearance of these autophagic vacuoles^29^. As an attempt to resolve this issue we examined the effect of IRI upon the ubiquitin binding adapter P62 (also known as SQSTM1). P62 binds to LC3 and ubiquitinates proteins and plays a key role in autophagy of ubiquitinated protein aggregates and organelles^30^,^31^. Our findings demonstrating that P62 was transcriptionally up-regulated in response to IR suggest that this treatment produced an increase in the number of autophagic vacuoles. P62 is a ubiquitin-binding protein demonstrated to facilitate autophagy- mediated clearance of autophagosome inclusions. Of particular relevance to the present studies is the remarkable work that by directly targeting P62 results in cargo loading failure and inefficient autophagy in the early stage, thus blocks cytoprotective autophagy of tumor cells to trigger apoptosis^32^.

**Figure 8:**
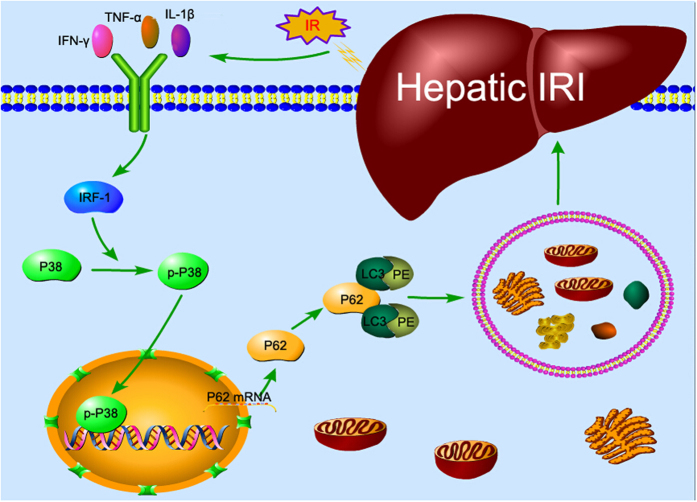
Model of IRF-1 aggravation of hepatic IRI resulting from P62 up-regulation induced increase in autophagy via P38-MAPK in hepatocytes. Figure 8 was drawn using Pathway Builder Tool 2.0 (http://external.informer.com/proteinlounge.com/).

In the present study, we found that IRF-1 expression was up-regulated in response to hepatic IR and accompanied by a corresponding activation of autophagy via P38/P62 activation (Fig. 8). To evaluate whether IRF-1 can aggravate hepatic IRI, we manipulated IRF-1 expression levels in our mouse models. IRF-1 expression was up-regulated by injection of AdIRF-1 via the caudal vein and down-regulated by knocking out the IRF-1 gene. IRF-1 overexpression significantly aggravated histopathological damage in livers following IR treatment^13^,^16^, whereas an IRF-1 deficiency exerted an opposite effect^33^. Cell viability was significantly decreased as a function of time following reperfusion in mice with an overexpression of IRF-1, whereas an IRF-1 deficiency significantly increased cell viability.

Related to these findings, was a significant increase in P38 activation after exposure to IR in livers overexpressing IRF-1. A considerable amount of evidence has been presented which demonstrate that P38 contributes to IR-induced liver damage, an effect which appears to result from the induction of apoptosis and necrosis^34^,^35^,^36^,^37^. In our study, we found that the increased degree of hepatocellular damage in mice resulting from overexpression of IRF-1 could be mitigated by SB203580 via inhibiting the activation of P38. These results indicate that IRF-1 decreased the viability of hepatocytes induced by IR via activation of the P38 signaling pathway.

The stimulation of P38 signaling transcriptionally up-regulates P62 expression, which plays an essential role in the generation of ALIS, the substrates for autophagy in macrophages. P62 is an important modulator in the network involved with the formation and degradation of the polyubiquitin-containing bodies for autophagy. Results from previous studies have shown that P62 is essential for the formation of LC3 structures^38^,^39^. To evaluate if the accumulation of P62 protein was the result of increased P62 transcription, we quantified P62 mRNA levels using qRT-PCR. Our results indicate that this accumulation of P62 protein was largely the consequence of an up-regulated transcription of P62. A ROS-P38-Nrf2 pathway has been recently found to be responsible for the maintain of high P62 protein levels and the formation of ALIS^40^. Several reports have disclosed that oxidative stress conditions results in the activation of Nrf2 and expression of its target genes, such as P62^41^. It leads to a transcriptional activation of Nrf2 through the P38-dependent mechanism. In line with this, blocking P38 MAPK reduced Nrf2 induction, increased ROS levels, and resulted in a reduced ubiquitinated-aggregate formation^42^.

LPS-induced ALIS formation is dependent on P62 levels, and knockdown of either of these molecules (such as MyD88, IRAK4, TRAF6) decreases LPS-induced GFP-LC3^+^ dot formation^22^. Next, to identify the kinases responsible for P62 induction, we focused on P38, which is a downstream kinase activated by TRAF6^43^,^44^. In the present study, we found that IR facilitated P38/P62 activation in livers leading to the accumulation of LC3II and autophagic vacuoles. Such a result is supported by other reports which have shown an accumulation of P62 or ALIS in cells exposed to chemically-induced oxidative-stress conditions^42^,^45^. Treatment with P38 inhibitor, SB203580, reduced this IR-induced P62 mRNA expression. Treatment with P38 inhibitor also reduced IR-induced P62-LC3 dot formation and the accumulation of ubiquitinated proteins in the detergent-insoluble fraction. These results suggest a role for P38, in IRF-1-mediated induction of P62 and ALIS formation.

## Materials and Methods

### Animals

Both C57BL/6 wildtype (IRF-1 WT; IRF-1^+/+^) and C57BL/6 background IRF-1-deficient (IRF-1 knockout [KO]; IRF-1^−/−^) mice were purchased from CasGene Biotech. Mice were maintained in a laminar-flow and specific pathogen-free atmosphere within the Laboratory Animal Center of Tianjin Medical University. Animal experiments were conducted in adherence to the guidelines of the China Association of Laboratory Animal Care. The protocols were approved by the Committee on the Ethics of Animal Experiments of Tianjin Medical University.

### Reagents

Dulbecco’s modified eagle’s medium/F-12 (DMEM/F-12, 12400) and fetal bovine serum (FBS, 10099) were purchased from Gibco. AdIRF-1 (adenoviruses encoding murine IRF-1) and AdGFP (empty adenoviral vector control) were purchased from Hanbio. IRF-1 siRNAs and siRNA-NC (negative control) were purchased from RiboBio Co., Ltd. (2131). SB203580 (S1076) was purchased from SelleckInc. Trizol (9108) and lipofectamine^TM^ 3000 (11668) were obtained from Invitrogen. Rabbit Abs specific for IRF-1 (8478), P38 (8690), p-P38 (4511), LC3 (12741), caspase-3 (9663), cleaved caspase-3 (9664) and horseradish peroxidase (HRP)-conjugated anti-rabbit secondary antibody (7074) were purchased from Cell Signaling Technology Inc. Anti-P62 Abs (5114) for immunoblotting were from Cell Signaling Technology Inc and anti-P62 Abs (BA2849) for immunostaining were from Boster. Anti-PCNA Ab (BS5842) was from Bioworld Technology Inc. Anti-GAPDH Ab (TA-08) was from ZSGB-BIO.

### Hepatic IR model

Mice had free access to water but were food restricted on the night before surgery. Following anesthesia with sodium chloral hydrate (30 mg/kg, ip), a non-lethal model of segmental (70%) hepatic warm ischemia and reperfusion was administered as described previously^46^. After laparotomy, an atraumatic vascular clip was used to interrupt the blood supply to the median and left lateral lobes of the liver, resulting in a 70% hepatic ischemia for 90 min. Reperfusion was initiated by removal of the clip. Sham control mice underwent the same procedure without vascular occlusion. Mice were euthanized at the scheduled times (2, 6, 12, 24 h) after reperfusion. Blood and liver tissues were collected for future analysis.

For adenoviruses propagation experiments, 40 μl of either AdGFP (1 × 10^10^ pfu) or AdIRF-1 (1 × 10^10^ pfu) were injected into mice via the caudal vein. For P38 inhibition, SB203580 (2 mg/kg) was injected into the mice via intraperitoneal injection. Six hours after injection, mice were subjected to the IR treatment.

### Serum ALT levels

Blood samples obtained from IRI mice were certrifuged at 3000 rpm for 15 min. Supernatants were collected and assayed for serum ALT levels using a standard automatic biochemistry analyzer.

### Liver histopathology

Liver parenchymas were fixed in formalin, embedded in paraffin, sectioned into 4-μm-thick sections, and stained with hematoxylin-eosin (H&E).

### Cell culture and treatment

The α-mouse liver 12 (AML12) cell line was purchased from the Institute of Biochemistry and Cell Biology of the Chinese Academy of Sciences. Cells were cultured in DMEM/F-12, supplemented with 10% FBS plus 100 U/ml penicillin and 100 μg/ml streptomycin (Solarbio).

IRF-1 was up-regulated by propagating AdIRF-1 into AML12 cells. For IRF-1 down-regulation, siRNA (F: 5′-GUAAGGAGGAGCCAGAAAU dTdT-3′, R: 3′-dTdT CAUUC CUCCUCGGUCUUUA-5′) was transfected into AML12 cells. SB203580 (0.5 μM, according to the reagent manual and data from preliminary experiments for targeting P38 MAPK) was added to the medium to inhibit P38 activation. Six hours after transfection, cells were transferred to a sealed pneumatorexis incubator to simulate IR conditions. The ischemia time was 90 min followed by 12 h-reperfusion.

### Immunofluorescence and Immunohistochemistry

Sections (4-μm-thick) were deparaffined and hydrated before antigen retrieval in 10 mM citric acid buffer. AML12 cells were inoculated in coverslips fixed in twelve-well plates. After rinsing with PBS, cells were fixed in 10% formalin for 30 min and incubated in 1% triton X-100 for 15 min. After eliminating endogenous peroxidase activity with 3% hydrogen peroxide in deionized water for 15 min, specimens were rinsed with PBS, then incubated with primary rabbit anti-P62 (1:200) or rabbit anti-PCNA (1:200) at 4 °C overnight. Specimens were washed with PBS and incubated with HRP-conjugated secondary antibody for 1 h on the following day. The conjugation reaction was facilitated by HRP-conjugated streptavidin and then visualized by diaminobenzidine (DAB, ZSGB-BIO). Images were obtained using bright-field microscopy.

### Transmission Electron Microscopy (TEM)

Livers and cell samples were placed in 1% glutaraldehyde and post-fixed with 2% osmium tetroxide. Sections or cell pellets were embedded in epon resin. The data were quantified by counting the number of autophagosomes per cross-sectioned cell.

### Confocal fluorescent microscopy

AML12 cells were cultured in 6-well plates to 60–70% confluence. To further detect autophagy induction, cells were transfected with tandem GFP-RFP-LC3 adenovirus (Hanbio) according to the instruction manual.

### Apotosis analysis

Apoptosis in liver sections was detected with the terminal deoxynucleotidyl transferase-mediated deoxyuridine triphosphate nick-end labeling (TUNEL) staining (Roche). TUNEL-positive cells were counted in 3 HPFs per section (×200).

### qRT-PCR

Total RNA was extracted by RNAiso Plus (Takara). The PrimeScript 1st Strand cDNA Synthesis Kit (Takara) was used to synthesize total cDNA in a 20 μl reaction system. According to the 2 × Taq PCR MasterMix real-time PCR kit (Aidlab), 1 μg cDNA was recommended as a template in the 25 μl reaction system of the PCR. The specific primers (Sangon) used were as follows: IRF-1 WT (F: 5′-TCCCATGTTCCAATGCTCGGT-3′, R: 5′-GCCCTTGT TCCTACTCTGATCCTTC-3′), IRF-1 KO (F: 5′-TCCCATGTTCCAATGCTCGGT-3′, R: 5′-AG GCATCCTTGTTGATGTCCCA-3′), P62 (F: 5′-AGGATGGGGACTTGGTTGC-3′, R: 5′-TCAC AGATCACATTGGGGTGC-3′), GAPDH (F: 5′-AGGTCGGTGTGAACGGATTTG-3′, R: 5′-T GTAGACCATGTAGTTGAGGTCA-3′). For RT-PCR, the reactions were performed on the 7000 Sequence Detection System (Applied Biosystems). Relative expressions were calculated as ratios normalized by GAPDH using the 2^−∆∆Ct^ method.

### Western blot analysis

Frozen liver tissue and AML12 cells were homogenized in RIPA lysis buffer and fully dissociated. The mixture was centrifuged at 12000 rpm for 15 min. The supernatant was collected. Protein concentrations were determined using the bicinchoninic acid (BCA) protein assay kit (Solarbio). Samples were mixed with loading buffer and run on a 12% sodium dodecylsulfate-polyacrylamide gel (SDS-PAGE). The proteins were transferred to polyvinylidene fluoride (PVDF) membranes. Membranes were blocked in 5% skim milk for 1 h and then incubated at 4 °C overnight with primary antibodies for IRF-1, P38, p-P38, Caspase-3, LC3, P62 and GAPDH. After being washed with TBST and incubated with HRP-conjugated secondary antibody for 1 h at room temperature, membranes were washed and developed with the Super Signal detection system (Pierce Chemical) and exposed to film.

### Small interfering RNA (siRNA) transfection

AML12 cells were cultured in 6-well plates overnight to 50–70% confluence. Lipofectamine^TM^ 3000 reagent was mixed into Opti-MEM containing siRNA1, siRNA2 and siRNA-NC, respectively, according to the manufacturer’s protocol. The mixtures were incubated at room temperature for 15 min and alliquoted into duplicate wells. Subsequently, cells were transferred to the incubator for 4–6 h to complete the transfection. The medium was replaced with DMEM/F-12 containing 10% FBS.

### Statistical analysis

Data are presented as mean ± SD. Comparison between two groups were performed by Student’s t test. For multiple comparisons, ANOVA was used. Statistical analysis was conducted by the GraphPad Prism software. A *P* value less than 0.05 was considered statistically significant. *P < 0.05, **P < 0.01, ***P < 0.001.

## Additional Information

**How to cite this article:** Yu, Y. *et al*. Interferon regulatory factor-1 activates autophagy to aggravate hepatic ischemia-reperfusion injury via the P38/P62 pathway in mice. *Sci. Rep.* **7**, 43684; doi: 10.1038/srep43684 (2017).

**Publisher's note:** Springer Nature remains neutral with regard to jurisdictional claims in published maps and institutional affiliations.
